# Adaptation of the Transverse Carpal Ligament Associated with Repetitive Hand Use in Pianists

**DOI:** 10.1371/journal.pone.0150174

**Published:** 2016-03-08

**Authors:** Christiane Mhanna, Tamara L. Marquardt, Zong-Ming Li

**Affiliations:** 1 Hand Research Laboratory, Department of Biomedical Engineering, Cleveland Clinic, Cleveland, OH, United States of America; 2 Department of Orthopaedic Surgery, Cleveland Clinic, Cleveland, OH, United States of America; 3 Department of Physical Medicine and Rehabilitation, Cleveland Clinic, Cleveland, OH, United States of America; Mayo Clinic Minnesota, UNITED STATES

## Abstract

The transverse carpal ligament (TCL) plays a critical role in carpal tunnel biomechanics through interactions with its surrounding tissues. The purpose of this study was to investigate the *in vivo* adaptations of the TCL’s mechanical properties in response to repetitive hand use in pianists using acoustic radiation force impulse (ARFI) imaging. It was hypothesized that pianists, in comparison to non-pianists, would have a stiffer TCL as indicated by an increased acoustic shear wave velocity (SWV). ARFI imagining was performed for 10 female pianists and 10 female non-pianists. The median SWV values of the TCL were determined for the entire TCL, as well as for its radial and ulnar portions, rTCL and uTCL, respectively. The TCL SWV was significantly increased in pianists relative to non-pianists (p < 0.05). Additionally, the increased SWV was location dependent for both pianist and non-pianist groups (p < 0.05), with the rTCL having a significantly greater SWV than the uTCL. Between groups, the rTCL SWV of pianists was 22.2% greater than that of the non-pianists (p < 0.001). This localized increase of TCL SWV, i.e. stiffening, may be primarily attributable to focal biomechanical interactions that occur at the radial TCL aspect where the thenar muscles are anchored. Progressive stiffening of the TCL may become constraining to the carpal tunnel, leading to median nerve compression in the tunnel. TCL maladaptation helps explain why populations who repeatedly use their hands are at an increased risk of developing musculoskeletal pathologies, e.g. carpal tunnel syndrome.

## Introduction

The transverse carpal ligament (TCL) is a band of collagenous tissue that constitutes the volar aspect of the carpal tunnel within the wrist. It inserts radially onto the scaphoid tuberosity and the ridge of the trapezium, and ulnarly onto the pisiform and the hook of the hamate. The TCL serves important roles in carpal tunnel mechanics by anchoring the thenar and hypothenar muscles [1, 2], functioning as a pulley for the flexor tendons [3, 4], and stabilizing the carpal bones in the transverse direction [5–7].

The unique anatomical configuration and biomechanical functions of the TCL may predispose the ligament to adaptations in response to repetitive hand use. It is known that soft connective tissues, including ligaments, remodel under mechanical loading conditions resulting in changes in their compositional and mechanical properties [8]. TCL remodeling may impose undesirable mechanical constraints on the carpal tunnel structure and its contents leading to pathological conditions implicating the median nerve, e.g. carpal tunnel syndrome (CTS). CTS is the most common upper limb entrapment neuropathy and has an increased incidence associated with repetitive hand use [9–11]. Adaptations of the TCL have been proposed as one of the possible etiological factors of CTS [12–15] and previous studies have anecdotally reported that CTS patients exhibit hypertrophy or stiffening of the TCL [13, 14]. However, the biomechanical changes of the TCL associated with repetitive hand use remain unclear and further investigation into such changes may elucidate the etiology of CTS, as well as contribute to understanding its prevention and progression.

Ultrasound imaging has been used as a clinical and research tool to provide noninvasive assessment of morphological and mechanical properties of soft tissues, including those of the TCL. For example, the TCL dimensions have been examined by A-mode [16] and B-mode [17] ultrasound imaging. Mechanical properties of the TCL have been studied using sonoelastography, which showed elevated TCL stiffness in patients with CTS [18]. Also, acoustic radiation force impulse (ARFI) imaging, a type of sonoelastography, has been used to assess the stiffness characteristics of the TCL in healthy individuals [19].

The purpose of this study was to examine the *in vivo* mechanical properties of the TCL associated with repetitive hand use using ARFI imaging. Pianists were selected as a model population because of their intensive piano playing involving the hands and the high incidence of musculoskeletal disorders (e.g. CTS [20, 21]). Playing piano generates biomechanical interactions among the TCL, thenar muscles, and flexor tendons, potentially inducing tissue maladaptations. It was hypothesized that pianists would exhibit a stiffer TCL than non-pianists. Furthermore, it was hypothesized that the increase in tissue stiffness would be location dependent, occurring at the radial aspect of the TCL where the thenar muscles responsible for thumb motion and strength are attached to the ligament.

## Methods

### Human Subjects

Twenty healthy, right handed female volunteers participated in this study. Ten of the participants were pianists (20.4 ± 1.6 years; BMI 22.5 ± 3.6 kg/m^2^) who practiced piano at least 10 hours per week for a minimum of 8 years (18.6 ± 14.9 hours per week for 12.8 ± 2.9 years). The remaining ten participants served as a control group (23.8 ± 3.4 years; BMI 20.5 ± 1.8 kg/m^2^) and were non-pianists. There were no statistical differences between the two groups in terms of phone texting (p = 0.423) and computer keyboarding (p = 0.165) based on their report of general, daily hand use. Exclusion criteria for the study included any history of upper extremity disorders or peripheral nervous system pathologies. The experimental protocol was approved by the Cleveland Clinic’s Institutional Review Board and each subject provided written, informed consent prior to study participation.

### Experimental Protocol

**Fig 1:**
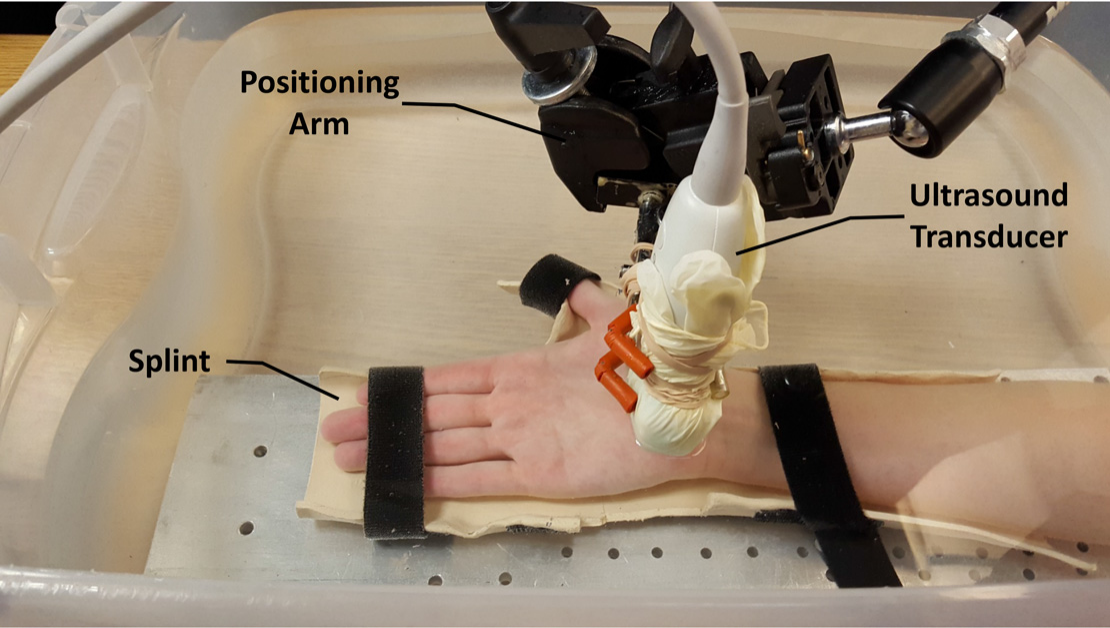
Experimental setup for acoustic radiation force impulse (ARFI) imaging of the transverse carpal ligament (TCL). Reprinted under a CC BY license, with permission from the Hand Research Laboratory at Cleveland Clinic, original copyright 2016.

Each participant sat next to a testing table with the hand and wrist stabilized in a supine, anatomically neutral position within a thermoplastic splint. The fingers, in full extension, and the thumb, abducted 0° palmarly and 45° radially, were secured with Velcro® straps. The stabilized hand and wrist were then submerged in a tank of room temperature water with the shoulder abducted 30° and the elbow flexed 90° (Fig 1).

ARFI imaging of the TCL was performed to measure shear wave velocity (SWV) using an ultrasound system equipped with Virtual Touch^TM^ Tissue IQ software (AcusonS2000, Siemens Medical Solutions USA, Inc., Mountain View, CA). A 9L4 linear array ultrasound transducer (Siemens Medical Solutions USA, Inc., Mountain View, CA) was used for image capture. The depth of the ultrasound image was set to 4.5 cm and the transducer was operated at an imaging frequency of 8 MHz.

A distance of 1.0 cm between the ultrasound transducer and the volar surface of the wrist was maintained to provide a water interface for ultrasound imaging that eliminated contact between the ultrasound transducer and the hand during experimentation. For each subject, image acquisition included simultaneously capturing a set of B-mode and ARFI ultrasound images that were displayed in a split screen format. The ultrasound transducer was oriented perpendicularly to the palm of the subject to capture transverse images at the distal level of the carpal tunnel. This location allowed for imaging of the TCL at its two distal osseous attachment points (ridge of the trapezium and hook of the hamate) along with the thenar muscles’ ulnar point (TUP). The TUP, which is the most ulnar aspect of the thenar muscles attachment to the TCL, is a unique anatomical feature that can be consistently identified on ultrasound images [17]. Four sets of images were captured and a single operator performed all imaging (CM). The transducer’s location and orientation were maintained throughout the experiment using a positioning arm. This protocol was implemented for the right and left hands of each subject.

### Data Analysis

**Fig 2:**
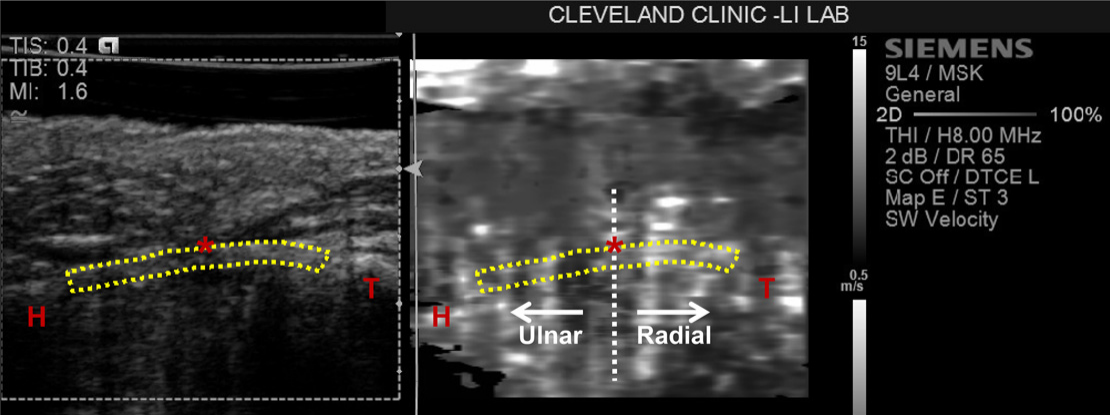
A representative ultrasound image with the TCL outlined on both the B-mode side (left) and ARFI side (right). The hamate (H), trapezium (T), and thenar muscles’ ulnar point (TUP,*) are identified. The vertical line at the TUP represents the location where the TCL was divided into radial and ulnar portions. Reprinted under a CC BY license, with permission from the Hand Research Laboratory at Cleveland Clinic, original copyright 2016.

A custom *MATLAB* (The Mathworks, Natick, MA, USA) program was used to complete data analysis. First, the TCL was manually traced on the B-mode image side. Then, this tracing was translated by the program to the ARFI image side (Fig 2). The SWV was calculated at each pixel within the TCL tracing, where the grayscale value (0–255) of each pixel in the ARFI image corresponded to a SWV value of up to 15.0 m/s. Within the TCL selection, the median SWV value was determined.

For further analysis, the TCL was divided into radial and ulnar portions at the TUP, i.e. rTCL and uTCL, respectively. The rTCL was defined as the region from the TUP towards the ridge of the trapezium, and the uTCL was the region from the TUP towards the hook of the hamate. The median SWVs for the rTCL and uTCL regions were also determined. Additionally, the ratio of the SWV of the rTCL to that of the uTCL was calculated.

### Statistical Analysis

A t-test was performed to compare the median SWV of the left and right hands of each subject. No differences were found between both hands, so the data was averaged across hands for each subject. An unpaired t-test was performed for group comparison of the SWV derived from the entire TCL without regional division. A mixed model, two-way repeated measures ANOVA was performed to investigate regional differences in SWV values using a between-subject group factor (pianist and non-pianist) and within-subject side factor (rTCL and uTCL). Post-hoc Tukey’s tests were used for all pairwise comparisons. All statistical tests were completed using SigmaStat 3.5 (Systat Software Inc, San Jose, CA, USA) with an alpha level of 0.05.

## Results

ARFI imaging revealed differences in the SWV of the TCL between pianists and non-pianists. Specifically, the SWV of the TCL was 10.2% greater for the pianist group than that for the control group with values of 5.52 ± 0.46 m/s and 5.01 ± 0.58 m/s, respectively (p < 0.05).

**Fig 3:**
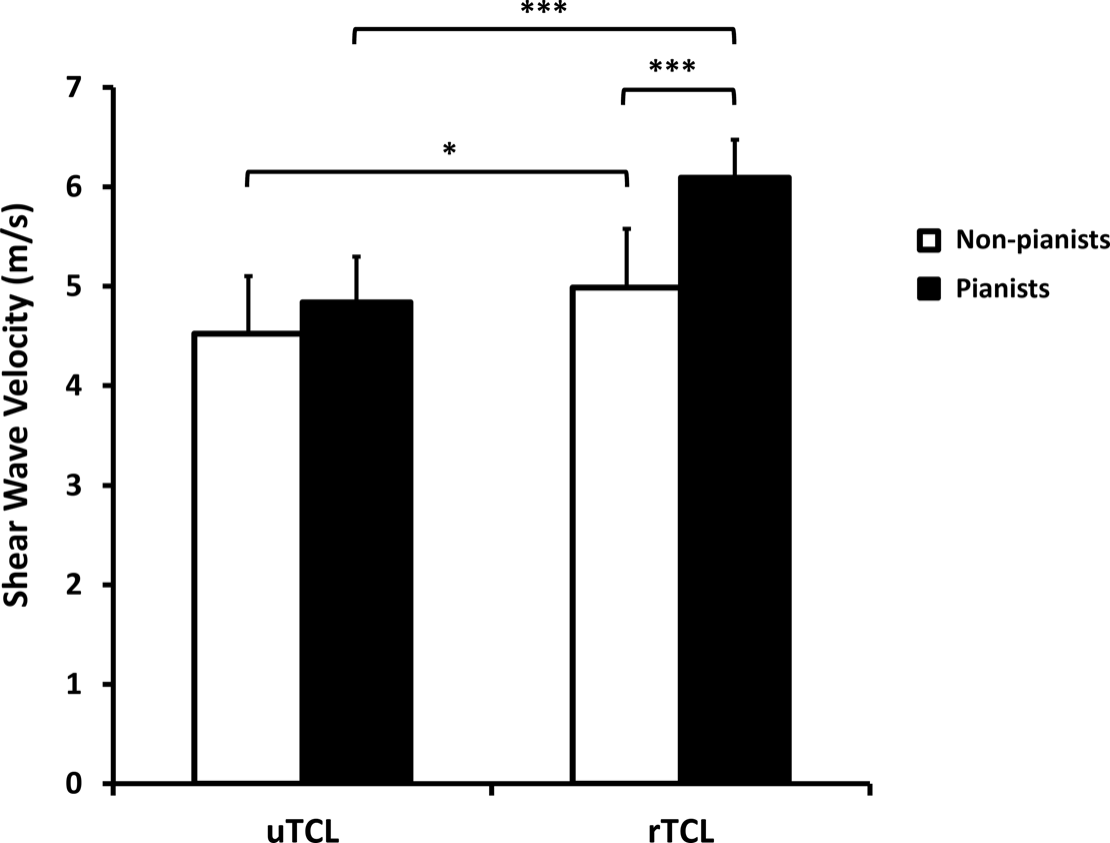
The shear wave velocities (SWVs) for the ulnar TCL (uTCL) and radial TCL (rTCL) in pianists and non-pianists. * p < 0.05, *** p < 0.001

Further analysis revealed that the SWV was significantly affected by factors of side (p < 0.001) and group (p < 0.05, Fig 3). There was also a significant interaction between the side and group factors (p < 0.01). Within the pianist group, the SWV of the rTCL (6.09 ± 0.63 m/s) was 25.8% greater than that of the uTCL (4.84 ± 0.38 m/s, p < 0.001). Similarly, there was a statistical difference between the SWV of the rTCL (4.99 ± 0.82 m/s) and the uTCL (4.52 ± 0.59 m/s) for the non-pianist group (p < 0.05).

Group comparisons showed that the SWV of the rTCL for the pianists was 22.2% greater than that for the non-pianists (p < 0.001), but the SWV of the uTCL was not significantly different between the two groups (p = 0.269). The SWV ratio of the rTCL to the uTCL for the pianist group (1.26 ± 0.14 m/s) was significantly greater than that for the non-pianist group (1.10 ± 0.10 m/s; p < 0.01).

## Discussion

ARFI imaging permitted *in vivo* quantification of the mechanical properties of the TCL by measuring the ligament’s SWV because a tissue’s SWV is positively correlated with the stiffness of the tissue [22]. The results confirmed the hypothesis that pianists have a stiffer TCL than non-pianists as indicated by a higher SWV. The stiffness changes in the TCL may be associated with the biomechanical interactions between the ligament and its surrounding tissues that occur during hand use. Further analyses also confirmed the hypothesis that the change in tissue property was location dependent, occurring only in the radial portion of the TCL but not in the ulnar portion.

Among many populations that partake in occupational and recreational activities involving repetitive hand use, pianists were chosen as a model for the study of tissue adaption due to the demanding biomechanical actions and tendon-TCL-muscle interactions associated with piano playing. The finding from this study that pianists have a stiffer TCL than non-pianists may help to explain the prevalence of CTS in pianists. Pianists are known to be at increased risk of developing CTS [20, 21], and the symptoms may be further exacerbated by playing-related technical faults, over practicing of parallel octaves, and exerting excessive pressure on the keys [23]. In the current study, it was found that even young, healthy pianists exhibited TCL adaptations, and it is possible that continued repetitive hand use may potentially result in tissue mal-adaptations and pathomechanical changes of the ligament leading to CTS.

Repetitive interactions between the TCL and its surrounding tissues may contribute to the changes in the ligament’s stiffness as found in the current study. During hand use, the TCL interacts with the flexor tendons located at its dorsal aspect and with the thenar and hypothenar muscles at its volar aspect. Although both groups showed that the stiffness of the rTCL was greater than that of the uTCL, the regional difference between the rTCL and uTCL was more noticeable for the pianists. The elevated stiffness on the rTCL for pianists may be attributed to increased biomechanical interactions of the thumb’s thenar muscles with the TCL associated with repetitive piano playing. It has been shown that 68% of the thenar muscles originate from the TCL [1], with the origin site on the radial aspect of the ligament. Therefore, thumb use with its associated thenar muscles contractions result in muscle-TCL interactions. Indeed, one of our previous studies revealed the biomechanical interaction among the TCL and thenar muscles during an isometric tip pinch and demonstrated that the TCL was pulled volarly with increasing magnitude as pinch force increased [24]. The action of piano playing involves repetitive abduction of the thumb for forceful depression of piano keys, which requires contraction of the thenar muscles. This increased focal biomechanical interaction between the muscles and TCL helps explain the location specific increase in SWV occurring at the radial portion of the TCL in pianists in comparison to non-pianists.

The results of this study should be interpreted with the consideration that only female subjects participated. Future studies may investigate if the findings in the current study are applicable to the male population. We expect that the biomechanical interactions among the anatomical structures and corresponding tissue adaptation take place regardless of gender. Another limitation is that the pianists who participated in this study had relatively large variability in weekly playing time as semiprofessional pianists, although their years of playing experience were similar. Even with the variation in playing hours, we were able to detect the tissue adaptation resulting from the repetitive hand activities. Future studies can be designed by including more homogenous pianist groups to investigate tissue adaptation in a dosage dependent manner.

In summary, ARFI imaging was used as a non-invasive tool to investigate the adaptations of the TCL associated with the repetitive hand use of piano playing. It was found that, in comparison to non-pianists, piano players have an increased SWV of the TCL, i.e. increased ligament stiffness. The stiffness of rTCL was greater than that of uTCL for both groups. However, the amount of difference between the rTCL and uTCL regions was greater for the pianists. This stiffening of the TCL may be primarily attributable to the biomechanical interactions that occur between the TCL and thenar muscles. Progressive stiffening of the TCL may become pathological, which provides an explanation for why populations who repeatedly use their hands have an increased incidence of CTS.

## Supporting Information

S1 File Subject-specific information and shear wave velocity (SWV) of the TCL are provided for non-pianists (Table A) and pianists (Table B) that underlie the main findings in this study. (XLSX)
